# Reduced Dorsal Visual Oscillatory Activity During Working Memory Maintenance in the First-Episode Schizophrenia Spectrum

**DOI:** 10.3389/fpsyt.2020.00743

**Published:** 2020-08-04

**Authors:** Brian A. Coffman, Gretchen Haas, Carl Olson, Raymond Cho, Avniel Singh Ghuman, Dean F. Salisbury

**Affiliations:** ^1^ Clinical Neurophysiology Research Laboratory, Western Psychiatric Hospital of UPMC, Department of Psychiatry, University of Pittsburgh School of Medicine, Pittsburgh, PA, United States; ^2^ Western Psychiatric Hospital of UPMC, Department of Psychiatry, University of Pittsburgh School of Medicine, Pittsburgh, PA, United States; ^3^ Center for Neural Basis of Cognition, Carnegie Mellon University, Pittsburgh, PA, United States; ^4^ Department of Psychiatry and Behavioral Sciences, Baylor College of Medicine, Houston, TX, United States; ^5^ Laboratory of Cognitive Neurodynamics, Department of Neurosurgery, Presbyterian Hospital, University of Pittsburgh School of Medicine, Pittsburgh, PA, United States

**Keywords:** first-episode schizophrenia, magnetoencephalography, visual short-term memory, working memory, alpha, gamma

## Abstract

Cognitive deficits in people with schizophrenia are among the hardest to treat and strongly predict functional outcome. The ability to maintain sensory precepts in memory over a short delay is impacted early in the progression of schizophrenia and has been linked to reliable neurophysiological markers. Yet, little is known about the mechanisms of these deficits. Here, we investigated possible neurophysiological mechanisms of impaired visual short-term memory (vSTM, aka working memory maintenance) in the first-episode schizophrenia spectrum (FESz) using magnetoencephalography (MEG). Twenty-eight FESz and 25 matched controls performed a lateralized change detection task where they were cued to selectively attend and remember colors of circles presented in either the left or right peripheral visual field over a 1 s delay. Contralateral alpha suppression (CAS) during the delay period was used to assess selective attention to cued visual hemifields held in vSTM. Delay-period CAS was compared between FESz and controls and between trials presenting one vs three items per visual hemifield. CAS in dorsal visual cortex was reduced in FESz compared to controls in high-load trials, but not low-load trials. Group differences in CAS were found beginning 100 ms after the disappearance of the memory set, suggesting deficits were not due to the initial deployment of attention to the cued visual hemifield prior to stimulus presentation. CAS was not greater for high-load vs low-load trials in FESz subjects, although this effect was prominent in controls. Further, lateralized gamma (34–40 Hz) power emerged in dorsal visual cortex prior to the onset of CAS in controls but not FESz. Gamma power in this cluster differed between groups at both high and low load. CAS deficits observed in FESz were correlated with change detection accuracy, working memory function, estimated IQ, and negative symptoms. Our results implicate deficits in CAS in trials requiring broad, but not narrow, focus of attention to spatially distributed objects maintained in vSTM in FESz, possibly due to reduced ability to broadly distribute visuospatial attention (alpha) or disruption of object-location binding (gamma) during encoding/consolidation. This early pathophysiology may shed light upon mechanisms of emerging working memory deficits that are intrinsic to schizophrenia.

## Introduction

People with schizophrenia have reduced capacity to maintain visual information in the focus of attention and working memory over short periods of time (1, 2). This deficit in visual short-term memory (vSTM), otherwise known as visual working memory maintenance (3, 4), may contribute to a variety of cognitive impairments in schizophrenia (5, 6). Working memory impairment is observable even at the first episode of psychosis (7, 8), and has been linked to functional outcome (9, 10). Neurophysiologically, people with schizophrenia show reduced activity during the delay between encoding and retrieval in frontal (11, 12) and parieto-occipital cortical areas (13, 14) when multiple items are held in vSTM. The mechanism of these impairments, however, remains unclear. Some have proposed that inefficiency of specific aspects of working memory performance, such as selective attention or consolidation, may account for observed vSTM deficits (13, 15). Although psychomotor processing speed is generally reduced in schizophrenia (16), covert attentional orienting/selection of stimuli during encoding is not delayed or reduced (17), ruling out problems in initial processing. Similarly, duration of the maintenance period does not seem to impact schizophrenia-related deficits in performance beyond about 1 s for visual stimuli (8, 18, 19), ruling out interference from distraction or increased decay of the percept. Thus, vSTM impairments in schizophrenia likely occur early in maintenance, possibly during consolidation of the percept.

A common method for assessing vSTM is visual change detection. In a typical change detection task, subjects maintain an image in working memory over a short delay period and indicate whether any item(s) in a later probe image has/have changed. The number of items presented (memory load) is manipulated and performance (e.g. *K*, an estimate of the number of items stored in memory) is compared between trials of varying load (3, 20). Although the task is quite simple, the outcome (*K*) depends on multiple factors. Poor performance could stem from errors during encoding, maintenance, or retrieval and could be related to poor orienting, feature selection/analysis, consolidation, accelerated decay of representations in working memory, interference from other sources of information, problems with executing an appropriate behavioral response, or any combination thereof. By directing attention to specific visual fields, some of these factors can be separated. In the lateralized change detection task, stimuli are presented peripherally, and participants are asked to remember the items from one visual hemifield while ignoring the other. This results in lateralized perceptual representations in posterior cortical areas not only during encoding, but also throughout the delay period, and concomitant lateralized neurophysiological responses measurable with magnetoencephalography (MEG). This enables comparison of neurophysiology between cortical hemispheres, providing a powerful within-subject control condition for measurement of group differences.

Contralateral alpha suppression (CAS) is the reduction from baseline of alpha band (8–12 Hz) spectral power contralateral to the focus of visual attention. CAS reflects the spatial focus of selective attention to external stimuli and to internally-generated perceptual representations (21, 22), making CAS a prime candidate for investigating selective attention during vSTM maintenance. Alpha oscillations in posterior cortex generally index the inhibition of ongoing neural activity (23), and may have a causal role in perceptual attention, with increased alpha in areas with inhibited processing of distractor stimuli, and reduced alpha activity in areas representing to-be-remembered perceptual activity. When applied externally *via* transcranial magnetic stimulation, perturbations in parieto-occipital alpha-band oscillations (but not beta- or theta-band) alter neural excitability, with diminished visual perceptual ability in visual field contralateral to the stimulated hemisphere, and increased ability in the ipsilateral visual field (24). In contrast, prior research on visual stimulus evoked oscillations suggests that low-frequency (LF) gamma band (30–40 Hz) spectral power these represents local excitatory/inhibitory network activity. Further, parieto-occipital LF gamma power perturbations contralateral to the attended visual stimulus are thought to index attentional perceptual mechanisms such as feature binding, scene segmentation, and/or stimulus representation (25). It is unknown if contralateral LF gamma power modulation can be identified within cortical generators of CAS or whether these phenomena are correlated. Gamma deficits in schizophrenia have been observed in a variety of contexts, including auditory (26, 27) and visual (28) sensation, and in frontal areas during working memory updating (29). However, effects of schizophrenia on contralateral parieto-occipital LF gamma power modulation during vSTM have not been investigated previously.

Reduced CAS during covert visual attention (30) and reduced (non-lateralized) alpha suppression during vSTM maintenance (22) have been reported in long-term schizophrenia. However, schizophrenia-related deficits in CAS have not been assessed during vSTM maintenance, and CAS deficits have not been reported early in the disease course. Here we used MEG to investigate CAS as a possible neurophysiological mechanism of vSTM impairment in people at first episode of schizophrenia-spectrum psychosis (FESz). This population is ideal for investigating cognitive deficits in early-stage schizophrenia, as medication effects have not yet become a confound for assessment of behavioral and neurophysiological responses. Further, to investigate whether cortical generators of CAS show co-localized and concomitant LF gamma power modulation, and given the long history and wide literature base showing deficits in selective attention (31–33) as well as disruptions in LF gamma oscillatory responses in both first-episode and long-term schizophrenia (27, 34–36), we examined broader spectral power differences in parieto-occipital sources of CAS.

## Methods

### Participants

Twenty-eight individuals with FESz and 25 HC participants were included in the study. Subjects were screened for colorblindness using pseudoisochromatic plates and had at least nine years of schooling as well as an estimated IQ over 85. None of the participants had: a) history of concussion or head injury with sequelae, b) history of alcohol or drug addiction or detox in the last five years, or c) presence of neurological disease or disorder. Participants provided voluntary informed consent and were compensated for participation. Procedures were approved by the University of Pittsburgh Institutional Review Board (IRB).

All participants completed the MATRICS Cognitive Consensus Battery (MCCB) (37), the Hollingshead Index of Socioeconomic Status (SES) (38) and the Wechsler Abbreviated Scale of Intelligence (WASI-I) (39). See **Table 1** for demographic measures. Research diagnoses were based on a consensus conference of baseline research assessment and confirmed 6 months after initial clinical assessment based on all longitudinal data at the 6-month follow-up assessment. Diagnostic status for all FESz and HC participants was based on findings from the Structured Clinical Interview for DSM-IV (SCID-IV) and consensus conference review. One FESz participant was lost to follow-up and therefore remains with the diagnosis of Schizophreniform Disorder (Provisional). Symptoms were rated using the Positive and Negative Symptom Scale (PANSS), Scale for Assessment of Positive Symptoms (SAPS), and Scale for Assessment of Negative Symptoms (SANS). All interviews and tests were conducted by an expert (Masters’- or PhD-level) clinical assessor (see **Table 2** for clinical measures). Of the 28 FESz participants, 18 received diagnoses of schizophrenia (paranoid: n=8; undifferentiated: n=8; residual: n=2), 4 of schizoaffective disorder (depressed subtype), and 6 of psychotic disorder not otherwise specified (NOS). All FESz participated within their first episode of psychosis and had less than 2 months of lifetime antipsychotic medication exposure. Eleven FESz (39.2%) were medication-naive.

**Table 1:** Participant Demographics and Neuropsychological Scores.

	Mean ± SD		*t*/χ^2^	*p*
	FESz	HC		
Sociodemographic data Age (years)	23.0 ± 4.8	21.6 ± 4.5	1.1	0.268
Sex (M/F)	18/10	16/9	0.1	0.983
Participant SES	29.6 ± 13.1	33.9 ± 15.2	−1.0	0.303
Parental SES	41.9 ± 13.6	47.9 ± 12.9	−1.6	0.117
Education (years)	12.6 ± 2.5	13.8 ± 3.1	−1.5	0.136
Neuropsychological Tests WASI IQ	107.9 ± 16.9	107.1 ± 9.4	−0.2	0.829
MCCB—Processing speed	42.5 ± 15.3	51.6 ± 8.3	2.7	**0.009**
MCCB—Attention	40.8 ± 11.5	46.2 ± 9	1.9	0.063
MCCB—Working memory	41.9 ± 14.5	46.8 ± 8.7	1.5	0.138
MCCB—Verbal learning	44.5 ± 11.2	51.1 ± 8.7	2.4	**0.020**
MCCB—Visual learning	40.6 ± 13	44.9 ± 8.1	1.4	0.157
MCCB—Reasoning	44.7 ± 12	50.9 ± 8	2.2	**0.030**
MCCB—Social cognition	45.6 ± 13.7	54.5 ± 9.1	2.8	**0.008**
MCCB—Total	38.6 ± 14.8	49.1 ± 6.7	3.3	**0.002**

Descriptive and inferential statistics are reported for first-episode schizophrenia subjects (FESz) and healthy controls (HC). Significant p-values are bolded. All other differences are non-significant (p > 0.05).

FES, first-episode schizophrenia; HC, healthy control; SES, Socioeconomic Status; WASI, Wechsler Abbreviated Scale.

**Table 2:** Patient Characteristics.

Symptoms	
PANSS—General	39.7 ± 6.8
PANSS—Negative	17.9 ± 4.9
PANSS—Positive	21.1 ± 5.1
PANSS—Total	78.7 ± 13.7
Medication data Cpz. equivalent dose (mg)*	218.6 ± 142.9
Medicated**/unmedicated	17/11

Descriptive statistics (mean ± SD) are reported for clinical variables and medication status for first-episode schizophrenia subjects.

** Of the 17 medicated participants, 13 were prescribed Risperidone, 5 were prescribed Olanzapine, and 2 were prescribed Aripriprazole (3 participants were prescribed two medications).

*Cpz equivalent dose is calculated only for medicated participants

PANSS, Positive and Negative Symptom Scale.

### Procedures

**Figure 1:**
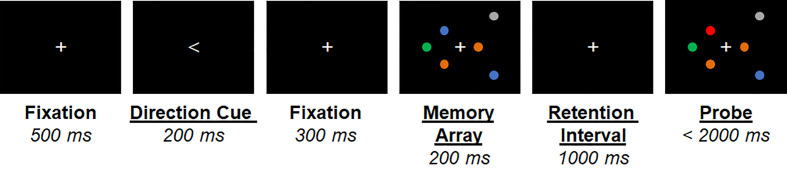
Graphical depiction of the lateralized visual short-term memory (vSTM) task. An example trial is shown for the attend-right high memory load condition. Timing of stimuli presented is shown below each stimulus.

Participants performed a lateralized change detection task (**Figure 1**). They were cued to covertly attend one visual hemifield (direction cue, 1.5° visual angle, 500 ms duration). An array of 1 (low-load) or 3 (high-load) filled colored circles was then presented in each hemifield (memory array) for 200 ms. One second later, another array was presented (probe) and participants indicated by button press with the right pointer or middle finger whether one of the circles in the attended hemifield had chane per hemifield). Participants were instructed to ignore changes in the unattended hemifield. The mapping of buttons (pointer/middle) to responses (change/no-change) was counterbalanced across participants. The following trial categories were equiprobable: no change, attended hemifield change, unattended hemifield change, or change in both hemifields. Thus, target responses (change/no-change) were also equiprobable. Participants had 2,000 ms to respond before the next trial. Circles could be one of 6 colors selected for equivalent luminance and color contrast. Circles subtended 0.65° and spatial locations were randomly selected from a 3° x 7° grid presented 1.5° to the left/right of central fixation. Stimuli were presented in five blocks of 75 trials, with short (~2 min) breaks between trials. The direction cue orientation, trial category, number of circles presented (low or high load), and spatial locations of the circles within the 3° x 7° grid were all randomly selected at the start of each trial.

### MEG

MEG data were obtained in a magnetically shielded room (Imedco AG, Hägendorf, Switzerland) using a a 306-channel whole-head MEG system (Elekta Neuromag) with a sampling rate of 1,000 Hz (online half-power band pass filter = 0.1–330 Hz). Bipolar leads were placed above and below the left eye (VEOG) and lateral to the outer canthi of both eyes (HEOG). Bipolar ECG leads were placed just below the left and right clavicle. Four head position indicator (HPI) coils were placed on the head and locations (relative to the nasion and preauricular points) were recorded using a 3D-digitizer (ISOTRAK; Polhemus, Inc., Colchester, VT). Head position was tracked continuously throughout the experiment.

### Structural MRI

Structural MRIs were obtained for use in MEG source modeling. Sagittal T1-weighted anatomical MR images were obtained using a Siemens TIM Trio 3 Tesla MRI system with a multi-echo 3D MPRAGE sequence [relaxation time/echo time/inversion time = 2530/1.74, 3.6, 5.46, 7.32/1260 ms, flip angle = 7°, field of view (FOV) = 220 x 220 mm, 1 mm isotropic voxel size, 176 slices, GRAPPA acceleration factor = 2].

### MEG Signal Preprocessing

The temporal extension of the Signal Space Separation method (40, 41) was used to remove noise sources outside of the MEG helmet and MEG sensor data were corrected for head motion using the Neuromag MaxFilter software (40). Using the MATLAB-based EEGLAB Toolbox (42), channels/segments with excessive noise or transient muscle artefacts were removed *via* visual inspection and a high-pass filter (0.5 Hz; 12 dB/oct) was applied. Adaptive Mixture ICA (AMICA) (43–45) was then performed to detect and remove one eye-blink and a maximum of 2 ECG components (representing pulsation and/or QRS artefacts) for each subject. Some subjects (N=5) did not present detectable ECG artefacts in the MEG signal. Components were identified based on their topography and temporal dynamics. All removed components were well-isolated (i.e. no additional blink/pulsation/QRS component was identified).

### MEG Analysis

The MEG sensor locations were registered to structural images using MRIlab (Elekta-Neuromag Oy, Helsinki, Finland). The locations of possible dipole sources were constrained to the gray/white matter boundary segmented from the structural MRI data using Freesurfer (http://www.surfer.nmr.mgh.harvard.edu) (46–48). This boundary was tessellated into an icosohedron with 5 mm spacing between vertices, resulting in a source model with ∼5,000 current locations per hemisphere. A forward solution was modeled as a single-shell (homogenous tissue) boundary-element model. The noise covariance matrix (calculated from the baseline interval of each trial) and forward solution were then used to create a linear inverse operator using a loose orientation constraint of 0.4 (0=current dipoles must be normal to the cortex; 1=no constraint) (49), with depth weighting applied. Continuous MEG data were filtered (100 Hz low-pass; 24 dB/oct) and source activity was then estimated from 204 planar gradiometer channels using MNE (50). After source modeling, correct trials were segmented from 300 ms before direction cue onset (i.e. 800 ms prior to memory array onset) to 500 ms after probe stimulus onset (i.e. 1,700 ms prior to memory array onset), and trials were rejected in which the magnetic field in any gradiometer exceeded 5 pT difference from baseline or eye movements were detected. Eye movements were detected in the HEOG channel using a step function, with a moving window or 200 ms duration with rejection criterion of +25 µV (51). Morlet wavelet deconvolution was then applied using 5 cycles at 1 Hz increments from 3 to 40 Hz, and event-related spectral perturbation was calculated as the relative change from baseline for each frequency measured. ROI analyses were performed on these native-space data, while vertex-wise analyses were performed on data morphed into a common space (fsaverage/MNI-305) with 10 mm smoothing.

Analysis of source-level time-frequency data then proceeded in a two-step analysis. First, to identify the generators of CAS, mean 8–12 Hz frequency power during vSTM maintenance (averaged from 200 to 1,200 ms after memory set onset) was assessed with vertex-wise one-sample t-tests across all subjects, separately for remember-left and remember-right trials. Parametric maps were corrected for multiple comparisons using spatial-cluster-based permutation testing with 1,000 iterations, using a cluster-forming threshold of *p*<0.05 and minimum cluster size of eight vertices (52). Regional labels corresponding to clusters of significant differences between high- and low-load were then identified from the Destrieux atlas implemented in Freesurfer. In the second step, average time-frequency maps were generated for each ROI, separately for each cortical hemisphere and stimulus condition. ROI-averaged time-frequency data ipsilateral to the attended hemifield were subtracted from those contralateral, and contralateral-ipsilateral difference spectra in high and low load conditions were compared between groups across the 3–40 Hz frequency spectrum and 200–1,200 ms retention interval time window using two separate time-frequency-cluster-based permutation tests (1,000 iterations, cluster-forming threshold of *p*<0.05, minimum cluster size of 8 contiguous time-frequency data points). Power within group-difference lateralized time/frequency cluster were then compared using separate 2 (group: HC vs. FESz) x 2 (load: high vs low) repeated-measures ANOVAs to identify possible group X load interactions.

### Demographic, Clinical, and Behavioral Data Analysis

Demographics and neurocognitive measures were compared between groups using independent samples t-tests and chi-square tests where appropriate. Task behavioral data including vSTM capacity (*K*) and reaction time (correct trials only) were subjected to a 2 (group: HC vs. FESz) x 2 (load: high vs low) repeated-measures ANOVA. *K* was calculated according to Rouder et al. (53) as the number of items to be remembered (*S*), multiplied by the ratio of the difference between hit rate (H) and false alarm rate (FA) to the correct rejection rate (1-FA), expressed as *K*=S*(H-FA)/(1-FA). Pearson correlations were computed separately for HC and FESz to explore relationships between behavioral and neurophysiological measures of working memory (K, RT, and contralateral-ipsilateral differential alpha/LF gamma power in high and low load conditions), and clinical/cognitive variables.

## Results

### Behavior

vSTM capacity (*K*) was lower in FESz than HC (*F*_(1,50)_=7.5; *p*=0.008; **Table 3**). An interaction between group and memory load (*F*_(1,50)_=4.7; *p*=0.035) was driven by greater vSTM capacity differences across groups at high memory load (*t*_50_ = 2.61; *p*=0.002) as compared to low load (*t*_50_ = 2.97; *p*=0.005). Differences between conditions were significant within both groups (*p’*s<0.001).

**Table 3:** Descriptive statistics for behavioral and neurophysiological effects.

	HC	FESz
K Low Load	0.95 ± 0.01	0.85 ± 0.03
High Load	2.19 ± 0.09	1.77 ± 0.13
Reaction time (ms) Low Load	725 ± 27	863 ± 30
High Load	822 ± 27	916 ± 31

Descriptive statistics (mean ± SEM) are reported for first-episode schizophrenia subjects (FESz) and healthy controls (HC).

FES, first-episode schizophrenia; HC, healthy control.

Response times were significantly slower for FESz compared to HC (*F*_(1,50)_=7.7; *p*=0.008), and for high memory load compared to low load (*F*_(1,50)_=99.3; *p<*0.001). Further, an interaction was found (*F*_(1,50)_=8.2; *p=*0.006), where differences between FESz and HC were greater at low (*t*_(50)_=3.3, *p*=0.002) than high (*t*_(50)_=2.2, *p*=0.03) memory load. The simple memory load effect was significant within both groups (*p’*s*<*0.001).

### Alpha Power

**Figure 2:**
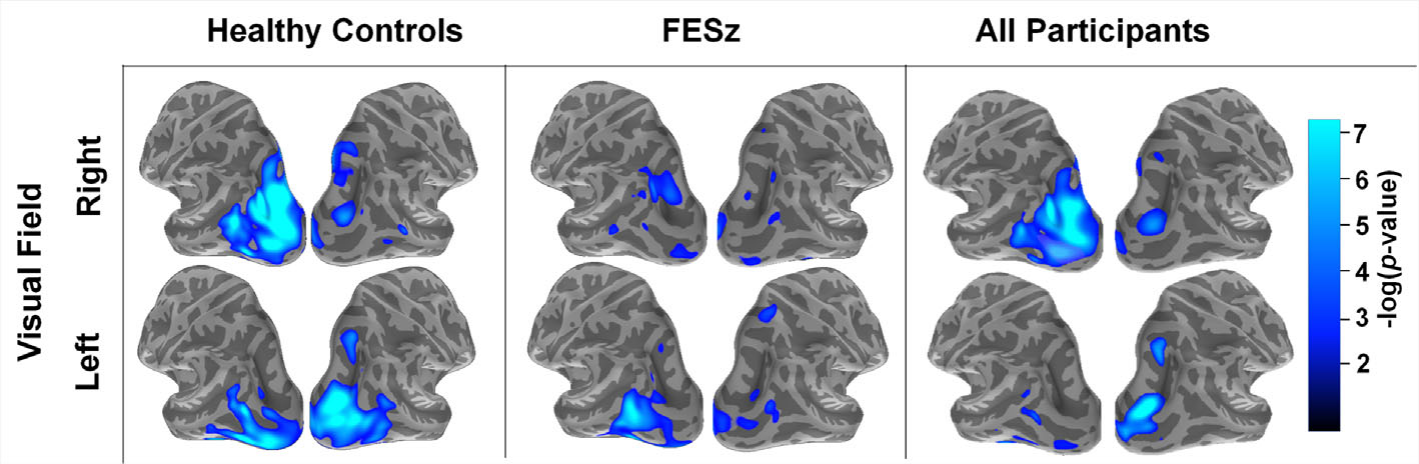
Spatial distribution of source-resolved contralateral alpha suppression (CAS) in high-load trials in healthy controls, first-episode schizophrenia (FESz), and all participants. Source-resolved activity is shown for stimuli presented in the right (upper) and left (lower) visual field. These images represent average alpha power between 8–12 Hz and 200–1,200 ms after the onset of the memory set stimulus, which was done as an initial analysis step prior to time-frequency cluster analysis (see **Figure 3**).

**Figure 3:**
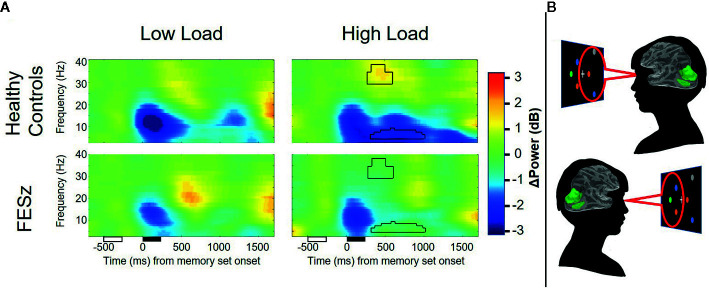
Average contralateral minus ipsilateral time-frequency spectra from dorsal occipital ROIs. Lateralized suppression of alpha and enhancement of gamma power during the maintenance period is evident in healthy controls, but not first episode schizophrenia patients. Unfilled and filled boxes below the x-axis represent onsets/durations of the attention cue and memory set stimuli, respectively. Power values are shown as relative change from baseline in decibels (dB). Time-frequency clusters surviving correction for multiple comparisons are depicted by black outline.

Mean alpha power during working memory maintenance was significantly reduced from baseline within dorsal lateral occipital cortex in both high and low memory load (**Figure 2**), with the most pronounced suppression in visual regions contralateral to the attended visual hemifield at high memory load. ROIs were generated in each hemisphere for the cortical vertices spanning the middle occipital sulcus, sulcus lunatus, superior occipital gyrus, superior occipital sulcus, and transverse occipital sulcus. Within average contralateral-ipsilateral difference spectra, only one time-frequency cluster was identified in the alpha band for high-load trials, spanning 7–11 Hz and 320–1,016 ms after onset of the memory array (**Figure 3**). No clusters were identified for low-load trials. Mixed methods ANOVA confirmed an interaction between group and memory load (*F*_(1,51)_=4.1; *p*=0.048; **Table 4**), which was driven by greater CAS in HC than FESz in high (*t*_(51)_=3.3, *p*=0.002), but not low load trials (*p>*0.1). Further, CAS was greater in high load than low load trials for HC (*t*_(24)_=2.5, *p*=0.021), but not FESz (*t*_(51)_=−0.1, *n.s.*).

**Table 4:** Descriptive statistics for spectral effects.

	CAS (dB)		Gamma Power (dB)	
	HC	FESz	HC	FESz
Low Load Left Visual Field	−0.41 ± 0.59	0.03 ± 0.64	0.53 ± 0.35	0.68 ± 0.29
Right Visual Field	−1.94 ± 0.55	−1.07 ± 0.76	0.11 ± 0.33	−0.22 ± 0.31
High Load Left Visual Field	−2.09 ± 0.66	−0.48 ± 0.56	0.29 ± 0.39	0.37 ± 0.32
Right Visual Field	−2.22 ± 0.61	−0.47 ± 0.57	0.73 ± 0.44	−1.17 ± 0.35

Descriptive statistics (mean ± SEM) are reported for first-episode schizophrenia subjects (FESz) and healthy controls (HC).

FES, first-episode schizophrenia; HC, healthy controls.

### LF Gamma Power Within CAS Clusters

A time-frequency cluster was identified in the LF gamma band spanning 34–40 and 276–600 ms after sample stimulus onset in high load trials, where contralateral LF gamma power increased from baseline in HC, but not FESz (**Figure 3**). No clusters were identified in low-load trials. ANOVA results did not indicate a group x memory load interaction (*p>*0.1). Rather, a trend-level main effect of group was found (*F*_(1,51)_=3.6; *p*=0.063; **Table 4**), indicating greater lateralized LF gamma power in HC vs FESz across memory load conditions. The main effect of memory load was not significant (*p>*0.1).

### Neurocognitive and Clinical Relationships

No significant correlations were found between oscillatory responses and reaction times; however, accuracy (*K*) was moderately correlated with CAS within high load trials in HC (*r* = −0.35; *p=*0.097) and FESz (*r* = −0.32; *p=*0.093). Additional correlations were detected only within FESz. Greater WASI IQ was significantly correlated with greater CAS in FESz (*r* = −0.41; *p=*0.028). Similarly, greater MCCB working memory scores were related to greater CAS in FESz for both high-load (*r* = −0.49; *p=*0.008) and low-load (*r* = −0.41; *p=*0.032) trials. The same was true for the reasoning and problem-solving scale (high-load: *r* = −0.42; *p=*0.025; low-load: *r* = −0.42; *p=*0.025). Among FESz, greater negative symptom severity (as measured by the PANSS) was related to reduced alpha for both high-load (*r* = 0.53; *p=*0.007) and low-load (*r* = 0.49; *p=*0.015) trials. After controlling for WASI IQ, these correlations remained significant except for correlations between CAS and MCCB reasoning/problem-solving scale, where magnitudes of *r*-values for low and high load trials were reduced to −0.35 and −0.39, respectively. No correlations were identified between CAS and LF gamma power, nor was LF gamma modulation correlated with any other variable (*p*’s>0.1).

### Medication Effects

As nearly half of our FESz sample was unmedicated, we compared behavioral performance (*K*, RT) and neurophysiological measures (CAS and lateralized LF gamma power) between medicated and unmedicated FESz participants to investigate the effects of medication. There were no significant differences between medicated and nonmedicated FESz on any of these measures, nor were any of these measures correlated with chlorpromazine-equivalent dosages in medicated patients (*p*’s>0.1).

## Discussion

Attentional control is fundamental to nearly all aspects of cognition, from learning and memory to social cognition and complex decision making (54–57). This is perhaps most evident in the link between attention and working memory. Attention and working memory are so tightly intertwined that some have even argued against a short-term memory storage system that exists separately from attention and long-term memory, suggesting that what we call working memory is actually a combination of attentional selection, rehearsal, and consolidation/off-loading to activated long term memory (56, 58, 59). Many others assert that attention cannot sufficiently explain all working memory phenomena observed and a separate system is needed for working memory, but still acknowledge the role of attention in controlling the processes of working memory (60, 61). In the current study, we examined the effects of schizophrenia on attentional control during working memory maintenance by investigating CAS, a robust and objective neurophysiological marker of the focus of attention.

Our results show reduced CAS in dorsal occipital cortex in FESz when multiple items are maintained in lateralized vSTM. CAS differences between groups begin ~300 ms after onset of the memory array and persist throughout the delay period in healthy subjects, but not FESz. This extends upon previous findings of reduced CAS during visual attention in long-term schizophrenia (30), and has strong implications for previous findings of reduced delay period activity, despite no difference in encoding and orienting of attention. Visual objects maintained in vSTM are quickly bound with their spatial locations into an internalized visual scene after sensation (~100–200 ms) and attention selection/orienting (~250–400 ms) (62). In this way, contextual aspects of the individual items can be registered and linked to representations in long-term memory (63, 64). During vSTM maintenance, this global context is assembled from the individual items held in working memory and itself maintained throughout the delay period. vSTM is facilitated when global patterns exist, suggesting that scene assembly during vSTM is adaptive (65). Delay-period CAS represents the focus of attention in this maintained visual scene (66). Our results suggest that something has gone awry with deployment of attention to multiple items during maintenance in schizophrenia. Indeed, individuals with schizophrenia are impaired in the ability to simultaneously attend to multiple locations (67), although the ability to narrowly focus attention to a single location is preserved (17) if not enhanced (68, 69). Deficits are observed in the shift of attention from global to local spatial context as well (70). Thus, individuals with schizophrenia-spectrum psychotic disorders may have limited access to global representations of objects maintained in vSTM due to reduced ability to distribute attention broadly, or due to disruption of object-location binding.

We also found that manipulating the number of items maintained in vSTM resulted in LF gamma-band oscillatory response in contralateral dorsal visual cortex, where LF gamma power increased for multi-object arrays in healthy controls. Interestingly, this LF gamma burst onsets before CAS, and shortly after attentional orienting. LF gamma power increase in dorsal occipital areas may indicate consolidation of visual objects in working memory and construction of internal scenes. Reported evidence of occipitoparietal LF gamma increases with unconscious learning of visual search arrays has similar timing to the effects observed here (71). LF gamma responses were absent in FESz at both low and high load here. Thus, we propose that FESz may have reduced ability to form visual scenes that can be searched in vSTM. This is concordant with previous findings of deficits in the allocation of spatial attention to multiple items (68, 72), as well as findings of reduced ability to shift between local and global context in visual scenes (70, 73, 74). However, it is important to note that we did not find that CAS was statistically correlated with LF gamma power modulation in this sample. It is also possible that gamma power increases spanned higher frequencies than those investigated here; however, we decided to focus on LF gamma power here following prior studies which have investigated wider-band frequency spectra in similar contexts (25, 71)

Although our results are consistent with literature showing reduced ability to focus broadly in individuals with schizophrenia compared to healthy controls, there are some inconsistencies between results observed in the low load condition here and what has been described in previous research. Specifically, Leanard, Luck, and colleagues (14) have shown larger responses in people with long-term schizophrenia compared to healthy controls in low memory load conditions, which has been interpreted as hyperfocusing on individual items during encoding and maintenance (68, 69). We did not find evidence of hyperfocusing here. It is possible that hyperfocusing may arise with progression beyond the early stage of the illness—we have investigated individuals at first episode of psychosis here and cannot speak to this hypothesis. Longitudinal studies are needed to determine the validity of this statement. Further, the study by Leonard et al. did not utilize the spatial cuing approach used here. Rather than using a directional cue to orient the participants prior to encoding, they used different shapes on either side of fixation. The spatial cue used in the current design may have given the opportunity for the control group to focus spatial attention before memory display onset more intensely than they would in a design without that forewarning. Indeed, deficits in cued redirection of covert spatial attention have been reported in long-term schizophrenia (75, 76), and controls here were more accurate than FESz at low load, which has not been reported previously.

In conclusion, our results show reduced lateralization of the focus of attention during vSTM, possibly due to impairment in the ability to broadly distribute spatial attention or reduced object-location binding. Forming hierarchical scene-based representations using visual information, semantic information, and objects is useful for organizing and summarizing information held in working memory. Healthy observers do this naturally in visual arrays, even when they do not explicitly contain patterns (65). Problems with the formation and/or utilization of scenes in vSTM could impact multiple aspects of schizophrenia, including visual learning, the ability to detect visual patterns (real or illusory), and the guidance of attention during social interaction. Deficits in the ability to represent high-level conceptual information along with object representations in working memory may prove fruitful for explaining complex aspects of working memory dysfunction that are hallmark of schizophrenia.

## Data Availability Statement

The raw data supporting the conclusions of this article will be made available by the authors, without undue reservation.

## Ethics Statement

The studies involving human participants were reviewed and approved by the University of Pittsburgh Institutional Review Board. Written informed consent to participate in this study was provided by the participants’ legal guardian/next of kin.

## Author Contributions

DS, RC, and CO designed the study and wrote the protocol. GH performed clinical evaluations. AG consulted on MEG source analysis. BC collected data and performed the data reduction and statistical analyses. BC and DS interpreted findings. BC wrote the first draft of the paper. All authors contributed to the article and approved the submitted version.

## Funding

This research was supported by funding from the National Institute of Health (P50 MH103204).

## Conflict of Interest

The authors declare that the research was conducted in the absence of any commercial or financial relationships that could be construed as a potential conflict of interest.
